# Respiratory support by neurally adjusted ventilatory assist (NAVA) in severe RSV-related bronchiolitis: a case series report

**DOI:** 10.1186/1471-2431-11-92

**Published:** 2011-10-20

**Authors:** Jean-Michel Liet, Jean-Marc Dejode, Nicolas Joram, Bénédicte Gaillard-Le Roux, Pierre Bétrémieux, Jean-Christophe Rozé

**Affiliations:** 1 Unité de Réanimation Pédiatrique, Hôpital Mère-Enfant Faïencerie, CHU de Nantes, 38 Boulevard Jean-Monnet, 44093 Nantes, France; 2 Service de Réanimation Pédiatrique, CHU de Rennes, 16 Boulevard de Bulgarie, 35000 Rennes, France

## Abstract

**Background:**

Neurally adjusted ventilatory assist (NAVA) is a new mode of mechanical ventilation controlled by diaphragmatic electrical signals. The electrical signals allow synchronization of ventilation to spontaneous breathing efforts of a child, as well as permitting pressure assistance proportional to the electrical signal. NAVA provides equally fine synchronization of respiratory support and pressure assistance varying with the needs of the child. NAVA has mainly been studied in children who underwent cardiac surgery during the period of weaning from a respirator.

**Case presentation:**

We report here a series of 3 children (1 month, 3 years, and 28 days old) with severe respiratory distress due to RSV-related bronchiolitis requiring invasive mechanical ventilation with a high level of oxygen (FiO_2 _≥50%) for whom NAVA facilitated respiratory support. One of these children had diagnosis criteria for acute lung injury, another for acute respiratory distress syndrome.

Establishment of NAVA provided synchronization of mechanical ventilatory support with the breathing efforts of the children. Respiratory rate and inspiratory pressure became extremely variable, varying at each cycle, while children were breathing easily and smoothly. All three children demonstrated less oxygen requirements after introducing NAVA (57 ± 6% to 42 ± 18%). This improvement was observed while peak airway pressure decreased (28 ± 3 to 15 ± 5 cm H_2_O). In one child, NAVA facilitated the management of acute respiratory distress syndrome with extensive subcutaneous emphysema.

**Conclusions:**

Our findings highlight the feasibility and benefit of NAVA in children with severe RSV-related bronchiolitis. NAVA provides a less aggressive ventilation requiring lower inspiratory pressures with good results for oxygenation and more comfort for the children.

## Background

Neurally adjusted ventilatory assist (NAVA) is a new method of assisted ventilation that can be used for children regardless of weight and age. This ventilation mode is controlled by diaphragmatic electrical signals through a gastric tube with specific electrodes on its surface. The collected electrical signals allow synchronization of ventilation to spontaneous breathing efforts of a child, as well as providing pressure assistance proportional to the electrical signal and thus to the output of the child's respiratory centers.

NAVA has mainly been studied in children who underwent cardiac surgery [1-3] during the period of weaning from a respirator. We report here a series of 3 children with severe respiratory distress due to respiratory syncytial virus (RSV) bronchiolitis for whom NAVA facilitated respiratory support.

## Case Presentation

Our unit is a 12-bed tertiary care university hospital pediatric intensive care unit. Recruitment is both medical and surgical. NAVA has been used in our unit for weaning children from a respirator who were operated on for congenital heart disease. The effectiveness of NAVA in these children led us to gradually expand the indications. We report a series of 3 children with severe respiratory distress due to RSV bronchiolitis for whom NAVA was used. The local ethics committee (*groupe nantais d'éthique dans le domaine de la santé *[GNEDS]) considered our report as non-interventional data research. The parents of all three children gave their written consent for publication.

Starting NAVA requires the initial correct positioning of the "NAVA" gastric tube. This commercially available feeding tube equipped with sensors (Edi catheter, Maquet Critical Care, Solna, Sweden) permits the recording of electrical activity of the diaphragm (Edi) via a Servo-I Ventilator (Maquet Critical Care, Solna, Sweden) using a standardized method [4]. Settings are relatively simple and include positive end-expiratory pressure (PEEP), fraction of inspired oxygen (FiO_2_), and level of NAVA assistance.

The Edi was multiplied was multiplied by the NAVA level to adjust the pressure assistance delivered to the child. The delivered pressure is equal to: NAVA level × (Edi max -Edi min) + PEEP. In clinical practice we usually started with a NAVA level of 1 cm H_2_O/μV that may have required adjustment if the Edi max signals deviated from a range between 5 and 20 μV. If the Edi signals turned out to be consistently greater than 20 μV, we increased the NAVA level until they are within this range. In the three reported cases, we did not need to do so.

During NAVA, the ventilator is triggered when the deflection in the Edi curve exceeds 0.5 μV. The assist is cycled-off when the Edi decreases to 70% of its peak value.

We assume that pressure support, which is pneumatically triggered, should remain a means of backup ventilation in case the Edi signal cannot be collected (e.g. if the child removes his/her Edi catheter). Therefore, we set the trigger of this pressure support high enough (typically 0 to -5 cm) so that this backup ventilation did not compete with NAVA ventilation.

We measured respiratory parameters (FiO_2_, tidal volume, mean airway pressure, peak inspiratory pressure, respiratory rate, and Edi max) directly from data exported from the respirator. Nurses recorded vital signs and SpO2. The oxygenation saturation index, OSI = (FiO_2 _× mean airway pressure)/SpO_2_, was used to provide a non-invasive method of oxygenation assessment. This index can be used for the diagnosis of acute lung injury (ALI) and acute respiratory distress syndrome (ARDS) in children when SpO_2 _values are ≤ 97% [5]. Diagnosis of ALI and ARDS required an acute onset of the process, bilateral infiltrates on a chest radiograph, no evidence of left atrial hypertension, and OSI > 6.5 (ALI) or > 7.8 (ARDS). Blood samples were also analyzed to provide blood pH and PCO_2 _values.

### Case 1

Shemsy was one month old (3.8 kg) without any particular risk factors. Her parents referred her to the emergency room because of grunting and hypotonia. She had rhinitis for several days with a cough for 48 hours following a viral contamination 3 days prior. She presented with apnea, desaturation, bradycardia and altered consciousness. She was intubated and ventilated immediately, and then transferred to intensive care.

Ten hours later, respiratory parameters were as follows: synchronized intermittent mandatory ventilation (SIMV) with a tidal volume (VT) of 20 ml (5 ml/kg); rate, 30/min; PEEP, 5 cm H_2_O; and FiO_2_, 50%. Measured parameters (stable for 2 hours) included a SpO_2 _of 91%, a mean airway pressure of 10 cm H_2_O, and a peak inspiratory pressure of 30 cm H_2_O (other parameters are also shown in Table 1). The OSI was 5.5. A chest X-ray showed poorly ventilated lungs with diffuse infiltrates. Since the child was agitated, the options for care were to increase sedation, or to attempt ventilation using NAVA. A brief test was undertaken to validate the use of NAVA, which proved successful. We chose to commence NAVA after a short period of decreased sedation (morphine was decreased to 8 μg/kg/h). Initial NAVA settings were PEEP, 5 cm H_2_O; NAVA level, 1 cm H_2_O/μV; and FiO_2_, initially 50% was then decreased by nurses to SpO_2 _> 90%.

**Table 1:** Ventilatory parameters (case 1)

	Before NAVA	Twelve hours after the start of NAVA
**Ventilation mode**	**SIMV**	**NAVA**

Peak inspiratory pressure (cm H_2_O)	30	(10)*

PEEP (cm H_2_O)	**5**	**4**

Mean pressure (cm H_2_O)	10	6

Tidal volume (ml/kg)	**5**	(4)*

Minute volume (ml/kg/min)	0.6	0.7

Respiratory rate (breaths/min)	**30**	(50)*

FiO_2_	**50%**	**21%**

SpO_2_	91%	92%

Edi max	< 5	(10)*

NAVA level	-	**1**

pH	7.38	7.41

PCO_2_, mmHg [kPa]	48 [6.4]	43 [5.7]

Data in bold are prescribed settings. Other data are measured parameters that depend on the ventilatory settings and the respiratory status of the child.

**Figure 1:**
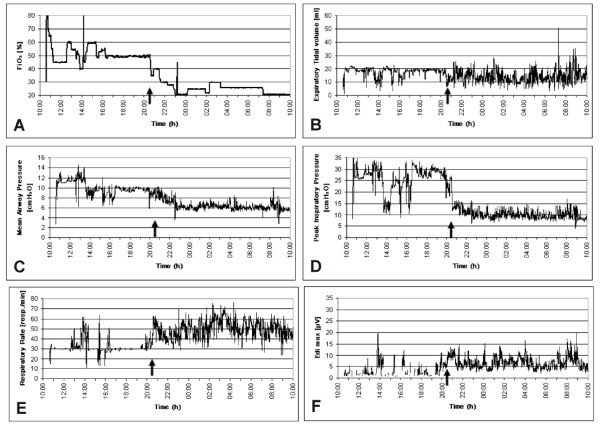
**Continuous recording of ventilatory parameters for 24 hours (case 1)**. Figure demonstrates in the first patient the 24-hour evolution of FiO2 (Panel A), expiratory tidal volume (Panel B), mean airway pressure (Panel C), peak inspiratory pressure (Panel D), respiratory rate (Panel E) and Edi max (Panel F). The upward vertical arrows indicate the time point where the ventilator mode was switched from SIMV over to NAVA. (A first brief NAVA period was tested at 14:00). Upper panels: After starting NAVA, (A) FiO_2 _gradually decreased to 21% in 12 hours, and (B) tidal volume became much more variable from one cycle to another. Middle panels: One of the most remarkable changes observed with switching to the NAVA mode was the immediate reduction in the mean airway pressure (C) and in the peak airway pressure (D) which decreased from 30 to 10 cm H_2_O. Bottom panels: After starting NAVA, the respiratory rate became very variable over time (E). From a mandatory frequency set at 30 breaths per minute, respiratory rate increased to 40 and 60 breaths per minute. Clinically, the breathing became easier with harmonious chest movements. (F) Edi max that it is the sum of inspiratory Edi and Edi min corresponds to the peak of electrical activity of the diaphragm. In SIMV, this activity is depressed, and in NAVA, the inspiratory Edi (Edi max - Edi min) drives ventilation. Abbreviations: FiO_2_, fraction of inspired oxygen; Edi, electrical activity of the diaphragm; SIMV, synchronized intermittent mandatory ventilation.

**Figure 2:**
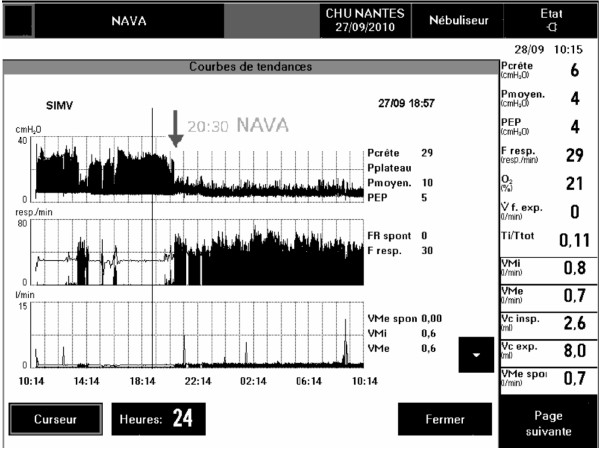
**Screenshot with trends over 24 hours (case 1)**. In the window untitled "Courbes de tendances" (Trend curves), three panels report trends over 24 hours of peak inspiratory pressure (cm H_2_O), respiratory rate (resp/mn), and minute volume (l/min). On the right of these panels, the values of these ventilatory parameters were collected to the vertical bar (at 18:57 while the child was not receiving NAVA). The downward vertical arrow indicates the switch from SIMV to NAVA (at 20:30). Outside the window untitled "Courbes de tendances", on the right of the scrennshot, ventilatory parameters were collected the next day at 10:15 while the child was receiving NAVA. The upper pannel showed a decrease in peak inspiratory pressure after the switch of ventilation. The middle pannel showed the extreme variability of the respiratory ratein NAVA (the white area under the curve corresponds to the mandatory respiratory rate, while the black area corresponds to the spontaneous respiratory rate). The lower pannel showed minute volume that remained unchanged. When comparing values of ventilatory parameters in SIMV (at 18:57) with those in NAVA (next day at 10:15), peak inspiratory pressure decreased from 29 to 6 cm H_2_O, mean airway pressure decreased from 10 to 4 cm H_2_O, spontaneous respiratory rate varied from 0 to 29 breaths/min. Abbreviations: SIMV, synchronized intermittent mandatory ventilation; *PEP*, positive end-expiratory pressure; *P crête*, peak inspiratory pressure; *P moyen*, mean airway pressure; *FR spont*, spontaneous respiratory rate; *F resp*, respiratory rate; *VM*, minute volume; FiO_2_, fraction of inspired oxygen.

* Data expressed in parentheses represent measurements that were very variable over time, and hence an estimate of the measured parameter is provided [see Figures 1 and 2].

Abbreviations: SIMV, synchronized intermittent mandatory ventilation; NAVA, neurally adjusted ventilatory assist; PEEP, positive end-expiratory pressure; FiO_2_, fraction of inspired oxygen; Edi, electrical activity of the diaphragm.

We observed a dramatic decrease in inspiratory pressure with a reduced requirement for oxygen (Figures 1 and 2). Within several minutes, the child's breathing became much more harmonious and smoother, while her respiratory parameters showed large variations from one cycle to another. SpO_2 _was96%, mean airway pressure was 6 cm H_2_O, peak inspiratory pressure was 10 cm H_2_O, and minute volume was 0.6 l/min.

Twelve hours later, FiO_2 _was decreased to 21% with a mean airway pressure of 6 cm H_2_O. Detailed ventilatory parameters are reported in Table 1. Since respiratory parameters were very low (Peak inspiratory pressure < 12 cm H_2_O with FiO2 < 25%) and blood gas values was normal, we extubated the child (10:30). She needed nasal continuous positive airway pressure for 3 days after which she was able to leave intensive care. Tracheal aspirate was positive for RSV and *Streptococcus pneumoniae*.

### Case 2

Matteo was 3 years old (14 kg), he was prematurely born (birth weight 1650 g) and he was mechanically ventilated in the neonatal period during 10 days for pulmonary hemorrhage.

He was recently hospitalized because of subcutaneous emphysema, with signs of acute respiratory failure from RSV infection. Because of an increased need for oxygen, he was intubated and ventilated with an FiO_2 _of 100%, and PEEP was 3. A chest X-ray did not show pneumothorax that could be drained. Tracheal aspirate was positive for RSV and *Hemophilus influenzae*. A few days later, because of emergence of an alveolar syndrome associated with persistence of subcutaneous emphysema, he was transferred to our unit for possible extracorporeal membrane oxygenation (ECMO).

Upon arrival, he had an oxygenation saturation index of 9.4 as well as with the other criteria for ARDS (bilateral infiltrates on a chest radiograph and no evidence of left atrial hypertension). Respiratory parameters were an SIMV with a VT of 85 ml (6 ml/kg), the rate was 25/min, PEEP was 6 cm H_2_O, and FiO_2 _was 70%. SpO2 was 89%, mean airway pressure was 12 cm H_2_O, peak inspiratory pressure was 28 cm H_2_O, and minute volume was 2 l/min. Venous blood gas showed a pH of 7.32, and a PvCO_2 _of 53 Torr (7.1 kPa). Because of a slight improvement, the child was not treated by ECMO. He underwent fibroscopy to eliminate the diagnosis of foreign body inhalation, which would have explained the subcutaneous emphysema. Forty-eight hours later, because of an increase in cough, cutaneous emphysema worsened and became diffuse (cervico-thoraco-abdominal) despite the reduction in PEEP to 4 cm H_2_O (FiO_2 _60%). We decided to use NAVA to improve the synchronization of mechanical ventilation with the child's spontaneous breathing. Sedation was reduced, midazolam was decreased to 80 μg/kg/h and sufentanil to 0.3 μg/kg/h. The child became alert. Initial NAVA settings were a PEEP of 5 cm H_2_O, NAVA level was 1.2 cm H_2_O/μV, and FiO_2 _was 60%.

**Figure 3:**
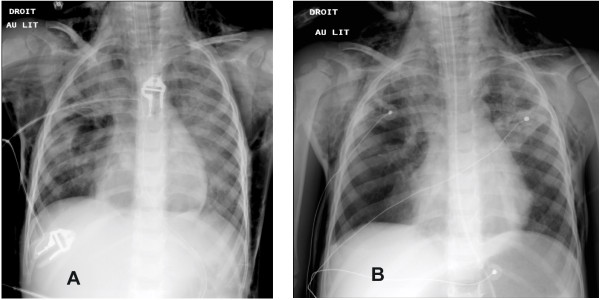
**Chest radiographs of case 2**. (A) The left X-ray shows bilateral infiltrates associated with diffuse subcutaneous emphysema. (B) Twenty-four hours after the onset of NAVA, the subcutaneous emphysema started to decline. Abbreviations: NAVA, neurally adjusted ventilatory assist.

This change reduced the requirement for oxygen and normalized blood gases (Table 2). The next day, the subcutaneous emphysema started to decline (Figure 3). Peak inspiratory pressure was between 10 and 19 cm H_2_O with an FiO_2 _of 50% for a SpO_2 _of 90%. The child's level of distress was scored with the modified COMFORT scale [6] and it ranged between 11 and 14 (adequately sedated, as confirmed by the child). This clear improvement allowed us to reassign the child to the original hospital 48 hours after initiation of NAVA. NAVA was continued at the second facility, permitting extubation 3 days later.

**Table 2:** Ventilatory parameters (case 2)

	Before NAVA	Twelve hours after the start of NAVA
**Ventilation mode**	**SIMV**	**NAVA**

Peak inspiratory pressure (cm H_2_O)	28	(15)*

PEEP (cm H_2_O)	**4**	**5**

Mean pressure (cm H_2_O)	12	7.5

Tidal volume (ml/kg)	**6**	(7)*

Minute volume (ml/kg/min)	2.0	3.1

Respiratory rate (breaths/min)	**25**	(30)*

FiO_2_	**60%**	**50%**

SpO_2_	89%	90%

Edi max	-	(10)*

NAVA level	-	**1.2**

pH	7.35	7.48

PCO_2_, mmHg [kPa]	64 [8.5]	43 [5.7]

Data in bold are prescribed settings. Other data are measured parameters that depend both on the ventilatory settings and the respiratory status of the child.

* Data expressed in parentheses represent measurements that were very variable over time, and hence an estimate of the measured parameter is provided.

Abbreviations: SIMV, synchronized intermittent mandatory ventilation; NAVA, neurally adjusted ventilatory assist; PEEP, positive end-expiratory pressure; FiO_2_, fraction of inspired oxygen; Edi, electrical activity of the diaphragm

### Case 3

Leane was 28 days old (3 kg) and was born at term. She had a 2-year-old brother with bronchiolitis. After an episode of rhinorrhea, she presented with feeding difficulties and was referred to the emergency room of a local hospital. Four hours later, she progressively presented with low oxygen saturation, tachypnea, and chest retraction. She was placed under nasal CPAP with 30% FiO_2_, and then transferred to our unit for severe RSV bronchiolitis. She was intubated on arrival because of clinical signs of respiratory distress and collapse. Although we suspected concomitant bacterial pneumonia because of a major inflammatory syndrome, we did not have bacteriological confirmation. Her respiratory status deteriorated rapidly. The OSI was 6.8 (with other diagnosis criteria for acute lung injury). Her need for oxygen increased rapidly with bilateral infiltrates on a chest radiograph.

**Figure 4:**
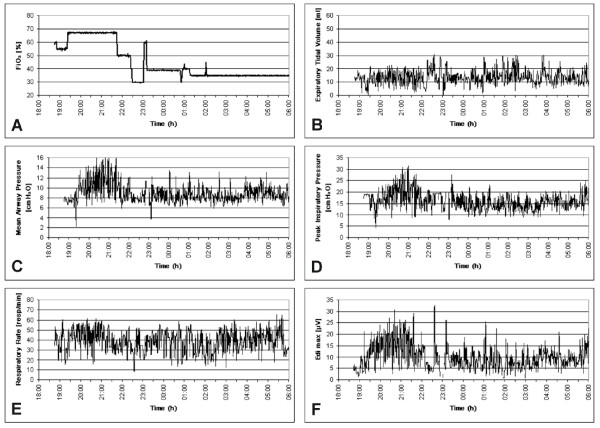
**Continuous recording of ventilatory parameters for 12 hours (case 3)**. Figure demonstrates in the third patient the 24-hour evolution of FiO2 (Panel A), expiratory tidal volume (Panel B), mean airway pressure (Panel C), peak inspiratory pressure (Panel D), respiratory rate (Panel E) and Edi max (Panel F). Recording began with the establishment of NAVA. Upper panels: After starting NAVA, (A) FiO_2 _gradually decreased to 35%, and (B) tidal volume became variable from one cycle to another ranging from 5 to 30 ml. Middle panels: Variability was also observed in measurements of pressure: (C) mean airway pressure, (D) peak inspiratory pressure. Bottom panels: (E) After starting NAVA, the respiratory rate became very variable over time. (F) Edi max corresponds to the peak of electrical activity of the diaphragm. The highest Edi values (recorded between 19:00 and 22:00) drove assistance with the highest pressures. A decrease in signal intensity was accompanied by a decrease in pressure, corresponding to an improvement in lung function. The requirement for oxygen decreased at the same time. Abbreviations: FiO_2_, fraction of inspired oxygen; Edi, electrical activity of the diaphragm; NAVA, neurally adjusted ventilatory assist.

We chose to use NAVA after a short period of decreased sedation (morphine was decreased to 8 μg/kg/h). Initial NAVA settings were a PEEP of 5 cm H_2_O, NAVA level was 1 cm H_2_O/μV, and FiO_2 _was initially 60% and was then adjusted by a nurse for a SpO_2 _> 90% and < 98%. Six hours later, FiO_2 _was 35%, while ventilatory pressures were lower than before starting NAVA (Table 3). As in the 2 other cases, ventilatory parameters were highly variable (Figure 4), while chest movements of the child were smooth as if the child was not mechanically ventilated. Twenty-four hours after starting NAVA, FiO_2 _was 21%. The modified COMFORT scale ranged between 7 and 13.

**Table 3:** Ventilatory parameters (case 3)

	Before NAVA	Six hours after the start of NAVA
**Ventilation mode**	**SIMV**	**NAVA**

Peak inspiratory pressure (cm H_2_O)	25	(15)*

PEEP (cm H_2_O)	**5**	**5**

Mean pressure (cm H_2_O)	10	8

Tidal volume (ml/kg)	**5**	(5)*

Minute volume (ml/kg/min)	0.6	0.8

Respiratory rate (breaths/min)	**40**	(40)*

FiO_2_	**60%**	**35%**

SpO_2_	88%	100%

Edi max	-	(10)*

NAVA level	-	**1**

pH	7.40	7.44

PCO_2_, mmHg [kPa]	52 [7.0]	47 [6.3]

Data in bold are prescribed settings. Other data are measured parameters that depend on the ventilatory settings and the respiratory status of the child.

* Data expressed in parentheses represent measurements that were very variable over time, and hence an estimate of the measured parameter is provided [See Figure 4].

Abbreviations: SIMV, synchronized intermittent mandatory ventilation; NAVA, neurally adjusted ventilatory assist; PEEP, positive end-expiratory pressure; FiO_2_, fraction of inspired oxygen; Edi, electrical activity of the diaphragm

At 36 hours of NAVA ventilation, the child was accidentally extubated during coughing. She immediately presented marked signs of respiratory distress (Silverman score: 7/10). We then used noninvasive ventilation with the NAVA option. Edi max values were initially very high (80 μV) and gradually decreased over 1 hour after the establishment of noninvasive ventilation. Thereafter, nasal continuous airway pressure was applied and the child left the intensive care unit 3 days later.

## Discussion

As in many pediatric intensive care units, our rate of intubation of children hospitalized for bronchiolitis is low (< 20%) [7]. However, the severity of lung disease in some children still necessitates invasive mechanical ventilation. Our unit recruitment is both medical and surgical, and we therefore acquired expertise in NAVA through the weaning of children treated after cardiac surgery. We then expanded the indications of this mode of ventilation in children with severe respiratory disease.

The positive results in the present study may be explained by the specific selection of children to whom we applied NAVA. First, there should be no specific contraindication to the placement of a nasogastric tube. Second, there should be no alkalosis or hypocapnia (in such cases there would not be sufficient diaphragmatic electrical activity). During alkalosis, the ventilatory brain centers no longer stimulate the diaphragm, and the respirator works on a backup mode, which is simply conventional ventilation. Finally, the level of sedation should not be too high, so that it does not depress brain centers that control breathing. If the sedation is too high, the Edi signal cannot be collected and the respirator works again in a backup mode. Likewise, neuromuscular connection from the respiratory center to the diaphragm must be intact. For example, NAVA cannot be used in case of post-surgical lesion of the two phrenic nerves [3] or diaphragmatic paralysis secondary to botulism [8].

The main benefit observed in our cases was an improvement in oxygenation associated with a normalization of blood pH. This was achieved with marked decrease in peak airway pressure. This effect has been previously found in crossover studies reporting NAVA in weaning children from a respirator who were operated on for congenital heart disease and comparing NAVA with pressure support [1-3]. Clinically, breathing becomes easier with harmonious chest movements. In one of our cases, NAVA was very effective for ventilation in a child who had both ARDS and extensive cutaneous emphysema. The excellent synchronization of mechanical ventilation with the spontaneous breathing of the child improved oxygenation without aggravating the emphysema.

Several factors could explain the beneficial effects observed with NAVA in these three children who had severe respiratory distress. First, asynchrony is associated with increased morbidity, a longer duration of ventilation, and a longer hospital stay [9-11]. There are few pediatric data published regarding the adverse effects of long-term asynchrony between mandatory ventilation and the respiratory efforts of children, but it has been shown that infant-ventilator asynchrony (both inspiratory and expiratory asynchrony) may affect more than 50% of the total breath duration [12]. Second, NAVA provides assistance in synchronization, as well as in pressure assistance in proportion to the measured electrical activity of the diaphragm. This helps to limit the periods of insufficient assist delivery that could induce respiratory muscle fatigue with increased oxygen consumption, and periods of over-assistance that can generate intrinsic PEEP with an inadequate increase in intrathoracic pressure [13]. NAVA can also prevent air swallowing and gastric distension by optimization of patient-ventilator synchrony [14]. Third, it is likely that NAVA can help clinician avoiding inappropriate ventilator settings that overload (or underload) respiratory muscles, preventing recovery. Finally, improvements in pulmonary gas exchange, systemic blood flow and oxygen supply to tissues which have been observed when spontaneous breathing has been maintained during mechanical ventilation with clinical improvement in the patient's condition [15], are assumed to occur in NAVA, when breathing efforts by the patient and the initiated breaths are in synchrony.

Cost effectiveness studies are required, as NAVA requires probes that are single-patient use. It is possible that improving comfort provided by better synchronization between spontaneous breathing and mechanical ventilation could reduce the sedation and thus shorten duration of ventilation.

## Conclusions

Based on three individual cases, NAVA appears to be a useful mode for weaning from a respirator and is an effective alternative treatment for children with severe respiratory distress. NAVA provides a respiratory support that is in harmony with the spontaneous efforts of breathing, allowing a decrease in inspiratory pressures and oxygen needs. Larger studies are required to compare NAVA with conventional respiratory support in children with various etiologies of respiratory distress.

## Competing interests

The authors declare that they have no competing interests.

## Authors' contributions

JML collected the data and drafted the manuscript. JMD participated in manuscript preparation and data analysis. NJ and BGL collected data and participated in the manuscript preparation. PB and JCR participated in the manuscript preparation and data analysis. All authors read and approved the final manuscript.

## Pre-publication history

The pre-publication history for this paper can be accessed here:

http://www.biomedcentral.com/1471-2431/11/92/prepub
